# Near-infrared spectroscopy cerebral oximetry in pediatric congenital heart disease with cardiopulmonary bypass: a narrative review of current evidence and neuroprotection

**DOI:** 10.3389/fped.2026.1822134

**Published:** 2026-07-08

**Authors:** Xiang Lin, Ling-Shan Yu, Qi-Liang Zhang, Lingqi Yu, Yi Zhang, Ye Chen, Zeng-Chun Wang

**Affiliations:** 1 Institute of Life Sciences, College of Biological Science and Engineering, Fuzhou University, Fuzhou, China; 2 Department of Cardiac Surgery, Fujian Maternity and Child Health Hospital, Fuzhou, China; 3 Department of Cardiac Surgery, Fujian Children's Hospital (Fujian Branch of Shanghai Children's Medical Center), Fuzhou, China; 4 College of Clinical Medicine for Obstetrics & Gynecology and Pediatrics, Fujian Medical University, Fuzhou, China

**Keywords:** brain injury, cardiopulmonary bypass, congenital heart disease, near-infrared spectroscopy, neurodevelopmental outcomes, regional cerebral oxygen saturation

## Abstract

Children with congenital heart disease (CHD) often require early surgical repair supported by cardiopulmonary bypass (CPB). Although survival has improved, neurological injury and later neurodevelopmental impairment remain common, motivating continuous perioperative neuromonitoring. Near-infrared spectroscopy (NIRS) provides noninvasive, real-time regional cerebral oxygen saturation (rScO_2_), reflecting the balance between cerebral oxygen delivery and metabolic demand. This review summarizes evidence on NIRS-derived perioperative rScO_2_ patterns in pediatric CHD surgery with CPB and examines associations between cerebral oxygenation abnormalities, markers of brain injury, and neurodevelopmental outcomes. rScO_2_ typically increases during cooling/deep hypothermia but reaches nadirs during low-flow perfusion or circulatory arrest and in early rewarming, suggesting vulnerability windows when oxygen supply–demand mismatch is most likely. Definitions and thresholds for cerebral desaturation vary substantially across studies, yet accumulating data indicate that postoperative cerebral oxygenation—particularly mean levels and cumulative desaturation burden within the first 12–24 h—may correlate with adverse biomarkers or neuroimaging findings and poorer later cognitive performance. Hemodynamic disturbance, oxidative stress, and inflammation may further shape the relationship between rScO_2_ abnormalities and neurological injury risk. Overall, perioperative rScO_2_ trends and desaturation burden may support neurological risk stratification and individualized physiological assessment, but standardized metrics, multimodal monitoring strategies, and prospective studies with long-term neurodevelopmental endpoints are needed to define actionable targets.

## 1 Introduction

**Figure 1:**
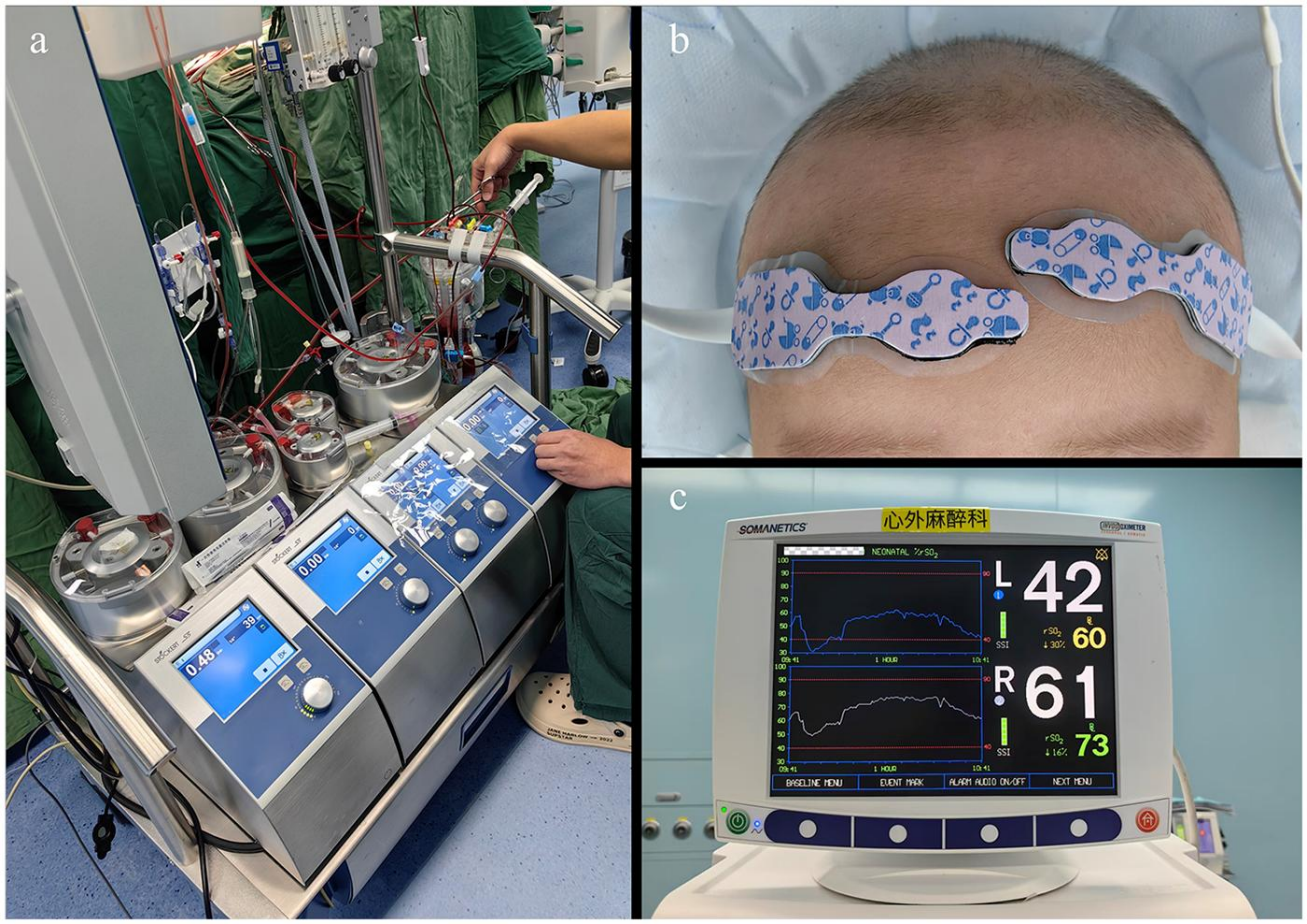
Schematic illustration of the CPB machine and NIRS device in the operating room during pediatric CHD surgery. **(a)** The CPB machine is positioned adjacent to the operating table and connected to the arterial and venous cannulas, providing circulatory and oxygenation support during surgery; **(b)** NIRS sensors attached to the patient's forehead for continuous monitoring of rScO_2_; **(c)** NIRS monitor displaying real-time cerebral oxygenation and related parameters. The two systems operate simultaneously, enabling real-time assessment of perfusion and oxygen delivery and informing intraoperative adjustments of anesthesia and perfusion settings. Source statement: The clinical photographs in Figure 1 were obtained from real intraoperative scenes in the hospital operating room. All clinical photographs were de-identified/anonymized before use, and no identifiable patient or healthcare personnel information is presented. Because no identifiable individual is shown, specific written informed consent for publication of these images was not required according to institutional policy.

Congenital heart disease (CHD) is one of the most common congenital malformations in children, with a global prevalence of approximately 1% of live births (1). Many complex CHDs, such as hypoplastic left heart syndrome (HLHS), transposition of the great arteries (TGA), and tetralogy of Fallot (TOF), typically require surgical repair during infancy, and cardiopulmonary bypass (CPB) is a key life-support technique that enables these procedures (Figure 1A). In recent years, advances in surgical techniques including CPB have markedly improved survival in children with CHD (2). However, neurodevelopmental outcomes among postoperative survivors have become a major clinical concern. Studies indicate that children with CHD undergoing CPB have a high risk of neurodevelopmental impairment, including mild deficits across multiple domains such as cognition, motor function, social skills, and executive function, with an estimated incidence of about 20%–60% (2).

The mechanisms of brain injury are complex and are closely related to multiple factors, including perioperative cerebral hypoperfusion, imbalance between oxygen delivery and consumption, and inflammatory responses. Therefore, during the perioperative period, particularly during CPB, real-time assessment and maintenance of the balance between cerebral oxygen delivery and metabolic demand are critical to reduce neurological injury and improve long-term neurodevelopmental outcomes. Multiple studies have examined the relationship between intraoperative and perioperative regional cerebral oxygen saturation (rScO_2_) and postoperative neurodevelopmental outcomes in children with CHD. Current evidence suggests that abnormal perioperative cerebral oxygenation patterns may be associated not only with short-term neurological injury but also with profound effects on mid- and long-term neurodevelopment. However, available studies remain limited and conclusions are heterogeneous (3–6). Accordingly, this narrative review aims to synthesize current evidence on NIRS-derived rScO_2_ in pediatric CHD surgery requiring CPB. The primary focus is on perioperative rScO_2_ trajectories, definitions and interpretation of cerebral desaturation thresholds and burden, associations between rScO_2_ abnormalities and brain injury or neurodevelopmental endpoints, and the limitations that currently prevent rScO_2_ from being considered an established therapeutic target. Mechanistic evidence on hypoxia, inflammation, oxidative stress, mitochondrial dysfunction, and CPB-related brain injury is discussed only when it helps contextualize rScO_2_ interpretation or supports future multimodal neuroprotective strategies. This review is therefore intended as a focused narrative review of cerebral NIRS/rScO_2_ monitoring rather than a broad review of all perioperative neuromonitoring methods, neurodevelopmental outcomes, or CPB-related brain injury mechanisms.

To improve transparency regarding literature identification and selection, this narrative review was based on a comprehensive but non-systematic literature search. The primary search focused on studies published from January 2015 to January 2026, while earlier seminal or methodologically important studies published from January 2000 onward were included selectively when they provided foundational evidence on CPB physiology, cerebral oximetry, cerebral perfusion strategies, or neurodevelopmental outcomes. The search primarily used PubMed and was supplemented by targeted searches in Web of Science, Google Scholar, and relevant publisher platforms, including Frontiers, Elsevier/ScienceDirect, SpringerLink, and MDPI, to identify recently published or methodologically relevant articles not readily captured by the primary PubMed search.

Search terms included combinations of “congenital heart disease”, “pediatric cardiac surgery”, “cardiopulmonary bypass”, “near-infrared spectroscopy”, “cerebral oximetry”, “regional cerebral oxygen saturation”, “rScO_2_”, “neurodevelopment”, “brain injury”, “MRI”, “biomarkers”, “cerebral autoregulation”, “CMRO_2_”, “cytochrome-c-oxidase”, “redox cytochrome-c-oxidase”, and “model-based NIRS”. We prioritized neonatal and pediatric CHD studies involving CPB and cerebral NIRS, studies reporting neurodevelopmental, neuroimaging, biomarker, or perioperative clinical endpoints, and relevant reviews, consensus statements, or guidelines. Adult, non-cardiac, and animal studies were included selectively when they provided mechanistic context or directly relevant emerging monitoring approaches. This review was intended as a narrative synthesis rather than a PRISMA-style systematic review or meta-analysis.

## 2 Monitoring of cerebral oxygen saturation during pediatric congenital heart surgery

Although CPB can maintain systemic perfusion and oxygenation, hemodilution, nonpulsatile flow, and the systemic inflammatory response induced by contact with the extracorporeal circuit may impair cerebral vascular autoregulation and alter the stability of cerebral blood flow, thereby increasing the risk of cerebral oxygen supply–demand mismatch and cerebral hypoperfusion (7–9). This risk is particularly prominent in neonates because cerebrovascular regulation is still developing, and they are prone to hypoperfusion- or hyperperfusion-related injury during hemodynamic fluctuations (e.g., low perfusion pressure during CPB) (9). In children with cyanotic CHD or single-ventricle physiology, preoperative cerebral oxygen delivery is often reduced, leaving insufficient physiological reserve to buffer intraoperative flow instability, thus, cerebral oxygen decompensation is more pronounced during critical CPB phases, increasing the risk of neurological injury.

In this high-risk context, perioperative cerebral oxygen monitoring is a key tool for real-time early warning of cerebral oxygen supply–demand imbalance and for guiding neuroprotective interventions during pediatric CHD surgery. The most commonly used modality for continuous cerebral oxygen monitoring is near-infrared spectroscopy (NIRS) (Figures 1B,C). NIRS is a noninvasive, continuous, real-time monitoring technique that leverages the scattering and absorption characteristics of near-infrared light (∼700–1000 nm) in tissue to quantify or semi-quantify oxyhemoglobin (HbO_2_) and deoxyhemoglobin (Hb), thereby estimating rScO_2_ in the subcortical tissue beneath the frontal cortex (10–12). Because the NIRS signal primarily reflects venous oxygenation, it better represents the balance between local oxygen delivery and consumption rather than serving merely as a surrogate for peripheral oxygen saturation or blood pressure (13). Accordingly, even when peripheral oxygen saturation and mean arterial pressure appear acceptable during CPB, NIRS can function as an early warning system for cerebral hypoperfusion risk (9). Compared with systemic indices such as blood pressure and arterial oxygen saturation, rScO_2_ more directly and sensitively reflects cerebral perfusion and cerebral oxygen supply–demand status; its dynamic changes during CPB can help identify vulnerable windows of insufficient cerebral oxygenation, providing a basis for timely neuroprotective intervention. Given its noninvasive and continuous nature, NIRS has been widely applied for perioperative cerebral oxygen monitoring in pediatric cardiac surgery.

Nevertheless, NIRS has limitations. For example, NIRS signals are subject to extracranial contamination: changes in scalp blood flow, probe pressure, intraoperative positioning, and venous return can alter rScO_2_ readings without necessarily reflecting true intracranial oxygenation. Inducing extracranial ischemia or hypoxia can markedly reduce readings across multiple NIRS devices. Moreover, measurements are not interchangeable across devices due to differences in algorithms and optical path design, and adult sensors may be unsuitable for infants (14–18).

At the clinical level, thresholds for defining cerebral desaturation are typically derived from population statistics and may not be applicable to special populations such as neonates, low-birth-weight infants, or children with cyanotic CHD, whose tolerance may differ. Baseline rScO_2_ values also vary across CHD diagnoses and phenotypes even before surgery, such that a single universal absolute threshold does not exist (19, 20). Given extracranial contamination and device-specific algorithms, rScO_2_ is better suited for trend monitoring and within-patient comparisons; absolute-value comparisons across devices or studies should be interpreted with caution.

Therefore, cerebral oxygen assessment should rely on integrated multimodal monitoring. This approach combines near-infrared spectroscopy with electroencephalography (EEG), arterial blood gas analysis, and key hemodynamic parameters. Interpretation should be individualized according to preoperative baseline values and intraoperative hemodynamic conditions. Such an approach may allow more precise guidance of perioperative neuroprotection strategies. EEG provides information on cortical electrical activity, and quantitative EEG during specific surgical stages (e.g., deep hypothermic circulatory arrest) can predict the subsequent magnitude of cerebral oxygen desaturation, partially compensating for the limitation that NIRS reflects oxygenation but not electrophysiological activity (21). Combining EEG and NIRS helps capture electrophysiological activity and oxygen metabolic status simultaneously, thereby improving early identification of perioperative neurological injury risk (21). Furthermore, when cerebral hemodynamic fluctuations or microembolism risk is of concern, transcranial Doppler (TCD) monitoring can provide cerebral blood flow velocity and microembolic burden information, enabling a more comprehensive assessment of neuroprotection-related risks (22).

## 3 Perioperative dynamics of cerebral oxygen saturation in children with CHD

### 3.1 Trends in cerebral oxygen saturation across CPB stages

Different phases of CPB can markedly affect cerebral rScO_2_. Temperature management is also an important determinant of rScO_2_ fluctuations. Studies have identified two key periods during CPB when rScO_2_ is prone to decline to low levels: (1) low-perfusion phases (including circulatory arrest or ultra-low flow) and (2) the early rewarming phase (23). During the early on-bypass cooling phase, CPB provides well-oxygenated blood while hypothermia reduces metabolism; thus, cerebral oxygen delivery is relatively in excess and rScO_2_ tends to increase. During deep hypothermic maintenance or low-flow perfusion, rScO_2_ typically remains relatively high, but once ultra-low flow or circulatory arrest occurs, rScO_2_ gradually decreases due to inadequate cerebral perfusion (21, 24–26).

With the onset of rewarming, metabolic demand increases rapidly. If cerebral blood flow and oxygen delivery do not rise in parallel, rScO_2_ is more likely to show a pronounced decline or increased variability during early rewarming (27). This may be related to a sharp increase in metabolic rate with temperature while perfusion has not yet adequately matched demand. As rewarming continues and perfusion flow is increased, rScO_2_ typically rises again. At the time of separation from CPB, rScO_2_ may decrease relative to the end-of-CPB level because extracorporeal support is removed while cardiac output is still recovering, but it usually remains above or close to the preoperative baseline (28). In the early postoperative period (within hours after CPB), rScO_2_ may decline further toward the normal range (28). Notably, in deep-hypothermia procedures, the late rewarming phase and the immediate period after weaning are critical for re-establishing cerebral oxygen balance, and sustained low rScO_2_ during this period warrants close attention. Rewarming is considered one of the most vulnerable windows for cerebral oxygenation, and recent studies suggest that slowing the rewarming rate may be a potential neuroprotective strategy (27, 29).

Overall, cerebral oxygen saturation during CPB exhibits a trend of initial elevation, subsequent decline and eventual recovery. It reaches the peak of elevation during the cooling and deep hypothermia phase, hits its nadir typically in the periods of low-flow, circulatory arrest and early rewarming, and tends to recover during normothermic weaning from CPB. These fluctuations reflect the real-time balance between cerebral oxygen delivery and consumption and suggest that anesthesia and CPB management should optimize cerebral perfusion during vulnerable periods—for example, ensuring adequate perfusion during low-flow phases and cautiously controlling rewarming speed—to avoid excessively low rScO_2_. In addition, oxygen delivery capacity influences cerebral oxygenation dynamics: initiation of CPB and priming-related hemodilution reduce hemoglobin concentration and arterial oxygen content, such that rScO_2_ may decrease even when pump flow appears stable (30). Pump flow, arterial partial pressure of carbon dioxide (PaCO_2_), and perfusion parameters jointly determine cerebral blood flow and oxygen extraction (31). Therefore, rScO_2_ should be interpreted in conjunction with hemodynamic variables (e.g., blood pressure, cardiac output) and blood gas indices (e.g., PaCO_2_, SaO_2_) to provide a reliable basis for clinical decision-making.

### 3.2 Cerebral rScO₂ trajectories during surgery across CHD diagnoses

Compared with healthy children, children with CHD often exhibit lower rScO_2_ even preoperatively. In complex cyanotic or single-ventricle CHD, baseline cerebral oxygenation is lower and cerebral perfusion is more unstable, making these patients more susceptible to cerebral oxygen supply–demand mismatch intraoperatively and postoperatively. For example, a prospective study reported that neonatal CHD patients had rScO_2_ values approximately 9 percentage points lower than healthy controls on average, and single-ventricle defects showed more pronounced cerebrovascular instability than biventricular defects (32). In duct-dependent CHD, cerebral oxygenation during the first 72 h after birth is generally low, with systematic differences across ductal-dependence subtypes (e.g., duct-dependent pulmonary circulation may have lower rScO_2_ than duct-dependent systemic circulation) (33). Distinct intraoperative rScO_2_ patterns have also been reported across CHD diagnoses. In general, preoperative baseline rScO_2_ is influenced by systemic oxygenation: cyanotic CHD (e.g., TOF, neonatal TGA) often involves chronic hypoxemia, leading to lower baseline rScO_2_ or near-normal values after compensation, whereas acyanotic lesions such as ventricular septal defect (VSD) tend to have near-normal baseline rScO_2_ (28). After initiation of CPB and cooling, rScO_2_ rises substantially in most patients. In one study involving several diagnostic groups, the preoperative rScO_2_ in the TOF group (71 ± 14%) was significantly higher than that in the TGA group (61 ± 11%) and VSD group (62 ± 9%), possibly because the TOF group received the highest inspired oxygen concentration during anesthesia, increasing arterial oxygen saturation. Approximately 10 min after cooling began, rScO_2_ in infants with TGA increased from ∼62% preoperatively to ∼90%, higher than the TOF (∼85%) and VSD (∼81%) groups at the same time point. During deep hypothermic maintenance and subsequent rewarming, TGA patients maintained the highest rScO_2_, with mean values significantly higher than those in the TOF and VSD groups. These findings suggest that neonatal cyanotic lesions such as TGA may demonstrate more prominent rScO_2_ increases during cooling, likely due to improved oxygenation from CPB and reduced metabolism under deep hypothermia, which mitigates preoperative hypoxemia. In contrast, acyanotic lesions such as VSD also show rScO_2_ increases during CPB but with relatively smaller magnitude (28). Another study also demonstrated that the core characteristic of non-cyanotic CHD is normal preoperative oxygenation, manifested by a high baseline rScO_2_. During CPB, the simultaneous decrease in mean arterial pressure(MAP) and Hb concentration directly disrupts the cerebral oxygen supply-demand balance. In the absence of compensatory mechanisms such as hypothermia to offset insufficient oxygen supply, rScO_2_ exhibits a significant downward trend. In contrast, cyanotic CHD is characterized by chronic preoperative hypoxia, presenting with a low baseline rScO_2_. During CPB, multiple factors mutually counterbalance each other: increased oxygen supply from elevated PaO_2_, reduced oxygen-carrying capacity due to decreased Hb concentration, and diminished cerebral oxygen metabolic rate induced by moderate/deep hypothermia strategies. These factors sustain the stability of the cerebral oxygen supply-demand balance continuously, thus resulting in no significant fluctuations in rScO_2_ throughout the entire process (34).

Overall, the rScO_2_ curve across surgical types is largely determined by whether deep hypothermia and circulatory arrest are involved. In simpler repairs or surgeries in older children, rScO_2_ changes are relatively modest. Meanwhile, in complex single-ventricle lesions such as HLHS undergoing the Norwood procedure, aortic arch reconstruction often requires deep hypothermic circulatory arrest (DHCA) or low-flow perfusion support (35). In such cases, the rScO_2_ trajectory is more complex: cerebral oxygen saturation increases during cooling but gradually declines during the arrest period when cerebral perfusion is interrupted, potentially approaching dangerous thresholds and necessitating close monitoring. To maintain cerebral oxygen supply, selective cerebral perfusion can be used during arch reconstruction to provide continuous head perfusion and avoid abrupt rScO_2_ decreases (36). After complex CHD surgeries such as the Norwood procedure, rScO_2_ upon ICU admission can be significantly lower than preoperative or intraoperative levels and may remain low for a period early after surgery (37, 38). Larger prospective studies further suggest that lower mean rScO_2_ or greater desaturation burden during the early postoperative period—especially within the first 48 h—is associated with an increased probability of lower overall IQ at 2-year follow-up, making the early postoperative window a key period for neuroprotection and intervention (39).

Across a longer perioperative trajectory, although peripheral oxygen saturation (SpO_2_) in cyanotic CHD patients usually improves rapidly after surgery, rScO_2_ does not necessarily increase accordingly and may remain similar to preoperative levels even before discharge. This suggests that improved systemic oxygenation does not immediately translate into improved cerebral tissue oxygenation, potentially due to factors such as increased postoperative metabolic demand, altered oxygen extraction, and redistribution of microcirculatory perfusion (40–42).

Differences across studies should be interpreted in the context of clinical and surgical heterogeneity. CHD subtype, cyanotic vs. acyanotic physiology, single-ventricle status, duct-dependent circulation, and surgical age all influence baseline cerebral oxygenation, cerebral oxygen extraction, autoregulatory maturity, and tolerance to hypoxic–ischemic stress (19, 20, 31–33). Recent neuromonitoring studies and reviews further emphasize that single-ventricle vs. biventricular physiology, preoperative cerebral oxygenation, monitoring timing, and perioperative confounders may affect interpretation of NIRS-derived cerebral oxygenation metrics (8, 12, 20). In addition, CPB/DHCA-related factors such as cooling depth, hematocrit, pump flow, selective cerebral perfusion, low-flow duration, and rewarming rate may substantially modify perioperative rScO_2_ trajectories (23, 26, 27, 35). These factors likely contribute to variability in reported rScO_2_ thresholds, nadirs, desaturation burden, and associations with neurological or neurodevelopmental outcomes.

## 4 Clinical significance and interpretation of cerebral desaturation thresholds

**Figure 2:**
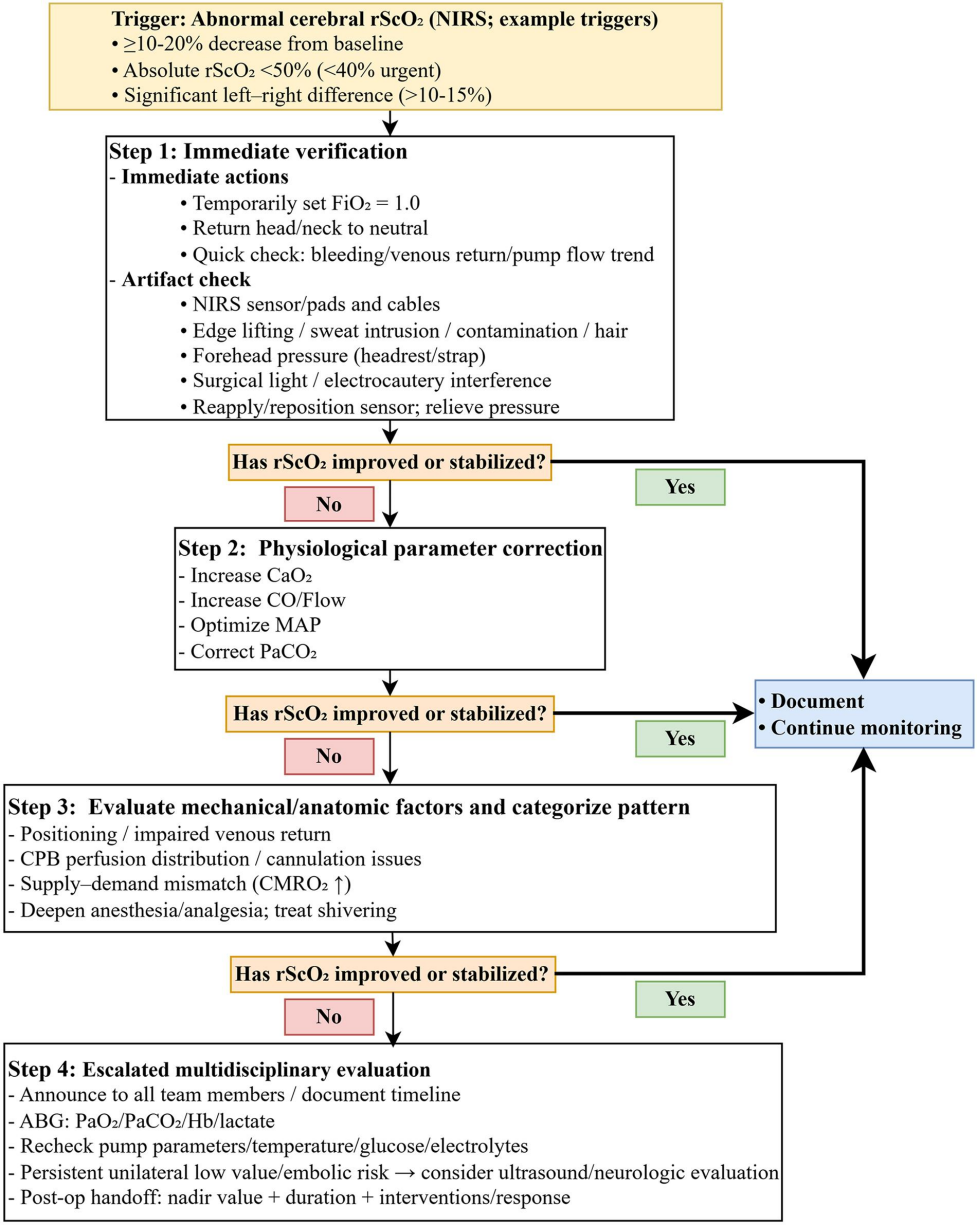
Conceptual, evidence-informed response framework for interpreting abnormal cerebral rScO_2_ during pediatric CHD surgery with CPB. The framework was developed by the authors to organize commonly reported physiological causes and bedside responses to abnormal cerebral rScO_2_ during pediatric cardiac surgery. It is not a validated clinical guideline, consensus algorithm, or universal management pathway. The trigger values shown in the figure, including a ≥10%–20% decline from baseline, absolute rScO_2_ < 50% or <40%, and marked left–right asymmetry, are examples derived from published cerebral oximetry literature and commonly used clinical warning thresholds; they should be interpreted according to patient-specific baseline rScO_2_, CHD subtype, cyanotic physiology, age, NIRS device characteristics, and CPB/DHCA conditions. The stepwise layout is intended to emphasize practical physiological assessment: first excluding artifact and immediately reversible causes, then optimizing oxygen delivery and perfusion, evaluating mechanical or anatomic contributors, and escalating evaluation when abnormal values persist. The framework should therefore be used as an interpretive aid for individualized assessment rather than as evidence that a specific rScO_2_-guided intervention strategy improves neurological outcomes. ABG, arterial blood gas; CaO_2_, arterial oxygen content; CMRO_2_, cerebral metabolic rate of oxygen; CO, cardiac output; FiO_2_, fraction of inspired oxygen; MAP, mean arterial pressure; PaCO_2_, arterial partial pressure of carbon dioxide. (created with draw.io, https://app.diagrams.net/).

Establishing cerebral desaturation thresholds in CHD surgery aims to convert the otherwise ambiguous state of cerebral oxygen supply into a quantifiable and actionable clinical standard. Early manifestations of cerebral ischemia or hypoxia are nonspecific, relying solely on routine parameters such as blood pressure and heart rate cannot accurately reflect cerebral oxygen metabolism. Moreover, clinicians' judgments about cerebral oxygen insufficiency may vary, leading to missed detection or false alarms. A threshold essentially serves as a critical warning line for cerebral oxygen supply–demand imbalance. When rScO_2_ falls to the threshold, it suggests that the brain is approaching an ischemic state but has not yet sustained irreversible injury. Initiating timely physiological assessment and supportive interventions at this point, such as optimizing perfusion pressure, oxygen delivery, or hemoglobin concentration, may help reduce the duration and severity of cerebral oxygen desaturation. Published cerebral oximetry studies have used heterogeneous warning criteria, including relative declines from baseline, absolute rScO_2_ thresholds, and desaturation burden, and no universally accepted threshold has been validated for all pediatric CHD populations (5, 12, 27, 43–46). Figure 2 therefore provides an evidence-informed conceptual framework for organizing the bedside assessment of abnormal cerebral rScO_2_ during pediatric CHD surgery with CPB. The trigger values and response steps reflect commonly reported cerebral oximetry warning thresholds and physiological response domains, but they are not intended as a validated guideline or consensus management algorithm. Their interpretation should be individualized according to baseline rScO_2_, CHD subtype, cyanotic physiology, age, device characteristics, and CPB/DHCA strategy (12, 19, 20, 23, 30, 31, 47, 48). Thresholds also help unify warning standards, transforming qualitative observation into quantitative decision-making and ensuring that all staff initiate interventions at the same trigger point, avoiding delays due to experience differences. The duration and magnitude of threshold-triggered desaturation can further serve as quantitative indices to evaluate the severity of intraoperative cerebral ischemia and to predict postoperative neurodevelopmental and cognitive impairment risk in children with CHD.

NIRS monitoring of frontal cerebral oxygen saturation has been widely used in perioperative management of pediatric CHD (12, 17). However, definitions of cerebral desaturation thresholds are inconsistent, limiting comparability across studies (5, 12). The literature generally describes three categories of thresholds: absolute thresholds, relative-to-baseline thresholds, and baseline-referenced time–depth composite metrics (5, 39, 49).

An absolute threshold is commonly defined as cerebral desaturation when rScO_2_ falls below a fixed value, such as <45% (5). Its advantage is intuitive and facilitates rapid clinical response; its limitation is that systematic offsets across NIRS devices, algorithms and baseline differences due to cyanosis or anemia can make a single absolute threshold overly sensitive or insufficiently sensitive. Reported absolute thresholds vary across studies, in part due to heterogeneity in monitoring technology and implementation details. NIRS readings can be influenced by probe placement, ambient light, and other factors, weakening cross-study consistency and comparability (12). rScO_2_ is also affected by device algorithms, scalp tissue properties, anemia, hemodilution, PaCO_2_, temperature, pump flow and blood pressure (46, 50–53), making universal absolute-value thresholds difficult to apply.

Rather than seeking a universal cerebral oxygen threshold applicable to all patients, a more practical strategy is to standardize the time point for baseline acquisition in both research and clinical practice (e.g., after tracheal intubation and before skin incision), incorporate baseline cerebral oxygen saturation into risk stratification, and use the magnitude of decline relative to baseline as an individualized warning signal. Among relative thresholds, the classic definition is a ≥20% decrease from baseline (5, 45). In infants, cerebral oxygenation may be more sensitive to changes, thus, a ≥10% decrease from baseline has been proposed as a warning threshold (43, 44).

Accordingly, an increasing number of studies have moved beyond whether the threshold was crossed and instead adopt baseline-referenced time-depth composite metrics that emphasize the duration and depth of hypoxia (44, 49, 54). Such metrics are more physiologically meaningful in characterizing hypoxic burden, although the lack of standardized calculation methods remains a major source of heterogeneity. Compared with the first two approaches, composite metrics may more robustly capture the association between perioperative hypoxic exposure and long-term neurocognitive outcomes. For example, Carra et al. found that greater desaturation burden and lower mean rScO_2_ were associated with lower total IQ at 2-year follow-up. This association remained consistent after adjustment for potential confounders such as disease severity (e.g., PIM3 score), persistent postoperative cyanosis, and nutritional strategies, supporting hypoxic burden rather than a single instantaneous threshold as a marker of long-term neurological risk (39). Therefore, threshold interpretation should account for patient-specific factors such as CHD subtype, cyanotic physiology, age, baseline rScO_2_, device characteristics, and CPB/DHCA strategy, rather than relying on a single universal cutoff.

## 5 Mechanistic context for interpreting cerebral oxygen saturation changes and neurological injury

Perioperative rScO_2_ reflects the balance between cerebral oxygen delivery and metabolic demand, but its clinical significance depends on the biological vulnerability of the developing brain and the systemic effects of CPB. In children with CHD, chronic preoperative hypoxia, immature cerebrovascular regulation, CPB-related hemodynamic disturbance, oxidative stress, inflammation, and mitochondrial dysfunction may jointly modify the relationship between cerebral oxygenation and neurological injury. Understanding these mechanisms helps interpret why similar rScO_2_ values or desaturation burdens may have different implications across CHD subtypes, age groups, and surgical strategies.

### 5.1 Developmental vulnerability driven by chronic cerebral hypoxia in children with CHD

The immature brain has distinctive pathophysiological susceptibility to hypoxic–ischemic injury, attributable to incomplete cerebrovascular autoregulation, high metabolic demand, and active myelination. Chronic hypoxia during critical periods of brain development (especially within the first 2 years when cerebral gray matter grows rapidly) can impair neuronal proliferation and differentiation. White matter development, particularly myelination, extends throughout childhood and adolescence. Hypoxic insults can impair oligodendrocyte maturation, leading to hypomyelination and subsequent white matter injury (55, 56). Clinical imaging studies have shown that 32% of infants with severe CHD have white matter injury on preoperative magnetic resonance imaging (MRI), which is associated with chronic fetal hypoxia (57). Delayed timing of surgery in CHD neonates may further prolong hypoxia, leading to cerebral oxygen metabolic imbalance and mismatch between cerebral blood flow and metabolic demand, ultimately triggering neurological injury such as periventricular leukomalacia (PVL) (58).

Reduced rScO_2_ is an important indicator of long-standing chronic cerebral hypoxia in children with CHD. Chronic preoperative cerebral hypoxia may trigger cellular metabolic adaptation, characterized by downregulation of mitochondrial respiration to reduce oxygen demand and avoid bioenergetic collapse (59). However, this compensatory adaptation can also suppress protein synthesis and cell-cycle progression, thereby affecting key neurodevelopmental processes including neuronal proliferation, neurogenesis, and myelination. The developing brain is particularly sensitive to mitochondrial dysfunction because immature mitochondria are more vulnerable to oxidative damage, excitotoxicity, and disturbances in energy metabolism (60, 61). Thus, chronic hypoxia not only constitutes preoperative functional abnormalities but also creates a highly vulnerable cerebral state at metabolic and structural levels in children with CHD.

### 5.2 Acute cerebral oxygen-metabolic perturbations mediated by CPB

Against the vulnerable background of chronic cerebral oxygen delivery insufficiency, perioperative CPB can induce acute disturbances in cerebral oxygen metabolism. Abnormal cerebral oxygenation after CPB primarily results from its direct effects on systemic hemodynamics, including increased cerebrovascular resistance, heterogeneous cerebral blood flow distribution, and mismatch between cerebral blood flow and metabolic demand. Systemic blood flow and oxygen transport parameters can substantially modulate cerebral oxygenation by influencing flow distribution and oxygen delivery efficiency (62, 63). In complex congenital heart disease, for example, in neonates with HLHS undergoing the Norwood procedure, prolonged DHCA and inappropriate CPB support strategies can further indirectly reduce oxygen delivery efficiency, increase the risk of hypoxic-ischemic injury, and lead to hypoxic-ischemic brain injury as well as adverse neurodevelopmental outcomes (64). Therefore, optimizing hemodynamics by modulating vascular resistance, optimizing PaCO_2_, and other approaches, while maintaining an appropriate hemoglobin level to improve oxygen delivery, can specifically alleviate cerebral hypoxia-ischemia, thereby reducing brain injury (65, 66).

Oxidative stress is considered one of the central mechanisms of brain injury after CPB and may impair neural cell function through reactive oxygen species (ROS) generation, activation of inflammatory signaling pathways, and disruption of redox homeostasis (67–69). Animal models (e.g., rats and piglets) have confirmed that CPB can lead to neurological and neurocognitive dysfunction (70), and can reduce mitochondrial oxidative phosphorylation in brain tissue; with longer CPB duration, mitochondrial ROS in brain tissue increases significantly (67, 71), indicating that CPB itself can induce cerebral metabolic imbalance and exacerbate oxidative neural injury. Animal studies also show that CPB suppresses neurogenesis in the subventricular zone; because the subventricular zone is the largest neural stem cell reservoir in the brain, impairment of this niche may directly affect cortical development (72). Clinical studies likewise show that after infant CPB surgery, urinary oxidative stress marker 8-iso-PGF2α and the serum brain injury marker s100 calcium-binding protein B (S100B) increase and are significantly associated with adverse postoperative neurodevelopment (73). Notably, a study in a neonatal piglet model demonstrated that hyperoxic CPB further increased cerebral ROS generation and reduced mitochondrial function, whereas controlled oxygenation (normoxia) during CPB did not significantly affect cortical mitochondrial function or oxidative injury in the acute phase (74). These results suggest that hyperoxic CPB is more likely than normoxic CPB to induce acute cortical mitochondrial dysregulation and oxidative injury; whether CPB-induced acute oxygen-metabolic perturbations are more prone to aggravate mitochondrial dysfunction in the context of chronic hypoxia remains to be further validated in chronic hypoxia models. Animal and small-sample neonatal studies suggest that allopurinol can attenuate hypoxia-reperfusion related brain injury by inhibiting ROS generation and has a favorable safety profile. A Dutch phase III randomized quadruple-blind multicenter placebo-controlled trial was initiated to investigate whether allopurinol improves brain injury in critically ill neonates with CHD requiring CPB cardiac surgery, while also evaluating potential cardiac protection and long-term neurodevelopmental effects with long-term follow-up(results have not yet been published) (75).

### 5.3 CPB-associated systemic inflammation and inflammation-mediated brain injury

Systemic inflammatory response syndrome (SIRS) is a common complication after pediatric congenital heart surgery and can substantially increase the risk of organ dysfunction (76, 77). In a cohort of 116 children undergoing CHD surgery, the incidence of SIRS within 72 h postoperatively was 34.5%, and it occurred almost exclusively in patients who received CPB (78). Direct contact of blood with artificial surfaces in the CPB circuit (oxygenator, tubing, membrane lung, connectors, etc.) can activate complement system, leukocytes and platelets, leading to neutrophil degranulation and the release of large amounts of proinflammatory cytokines such as IL-6, IL-8, and TNF-α, forming an inflammatory cascade involving both humoral and cellular components. Children, especially neonates and infants, exhibit a more pronounced CPB-induced inflammatory response. This may be associated with factors such as the relatively larger blood contact area of the extracorporeal circuit, hemodilution and allogeneic blood exposure related to perfusion, immature inflammatory regulation, and shear stress caused by small-caliber tubing under high target flow (79–81).

CPB-associated systemic inflammation can damage the central nervous system through multiple mechanisms. After inflammatory mediators reach the CNS, they can disrupt blood–brain barrier integrity, leading to cerebral edema and brain swelling and further aggravating cerebral hypoperfusion. Neuroinflammation can directly injure neural tissue and reduce tolerance to hypoxic–ischemic insults, ultimately contributing to reduced gray and white matter volumes, cortical thinning, and vascular injury (e.g., lacunar infarction) (82, 83). Inflammation often acts synergistically with microembolism and hypoperfusion, further exacerbating neuronal damage. Because the cerebral metabolic rate in neonates and infants is approximately 30% higher than in adults, this population is more sensitive to inflammatory stimuli and may sustain more severe injury (84).

Microglial activation is a key feature of CPB-induced neuroinflammation. Meng et al. systematically reviewed core signaling pathways involved in microglial activation, including MAPK, JAK/STAT, NF-*κ*B, Notch, PPARs and TLR pathways, which jointly regulate inflammatory responses, immune functions, and cell survival (82). In a juvenile piglet model, pathological changes after CPB included a sharp increase in microglial numbers, excessive activation of STAT3 signaling, intensified inflammatory stress responses, and markedly increased neuronal apoptosis, culminating in significant behavioral abnormalities. Delivery of bone marrow-derived mesenchymal stromal cells (BMSCs) via the CPB circuit provided neuroprotection by suppressing excessive STAT3 activation, reducing microglial activation, and alleviating inflammatory stress, thereby mitigating CPB-related neurological injury and improving behavioral abnormalities (85). These findings support microglia as a key therapeutic target for neuroprotection after CPB.

Several inflammation-targeted or mechanism-based interventions have been explored, but their clinical neuroprotective efficacy in pediatric CHD surgery with CPB remains unproven. For instance, dexmedetomidine has been reported to attenuate inflammation after cerebral ischemia and to exert neuroprotective effects on the hippocampus through α_2_-adrenergic receptor–related mechanisms, mainly in experimental or non-pediatric CHD settings (86, 87). However, evidence that it improves neurodevelopmental outcomes in infants and children undergoing CPB remains insufficient. Therefore, ongoing and future pediatric trials should be interpreted as evaluating a biologically plausible strategy rather than confirming an established neuroprotective therapy. Consistent with this rationale, dexmedetomidine has entered the design phase of a prospective randomized trial involving infants and young children undergoing CPB (87). Similarly, earlier studies suggested that nitric oxide might help mitigate SIRS in children undergoing CPB (88–90); however, a recent randomized controlled trial with 12-month follow-up reported that adding nitric oxide to the oxygenator during CPB for infant open-heart surgery did not improve neurodevelopmental outcomes or quality of life (91). These findings indicate that inflammation-targeted interventions remain investigational and require outcome-focused validation.

For cerebral NIRS interpretation, these inflammatory and microvascular mechanisms are relevant because they may alter cerebral perfusion, oxygen diffusion, metabolic demand, and tolerance to hypoxic–ischemic stress, thereby modifying the relationship between rScO_2_ changes and neurological outcomes.

### 5.4 The two-hit model and clinical implications

The two-hit model provides a conceptual framework for interpreting heterogeneity in the prognostic meaning of perioperative rScO_2_ abnormalities. Neurological injury in children with CHD is not attributable to a single perioperative event; rather, chronic fetal or preoperative oxygen-delivery impairment may create a vulnerable metabolic and structural brain state, while CPB-related inflammation, oxidative stress, and perfusion instability may reduce tolerance to subsequent rScO_2_ disturbances.

**Figure 3:**
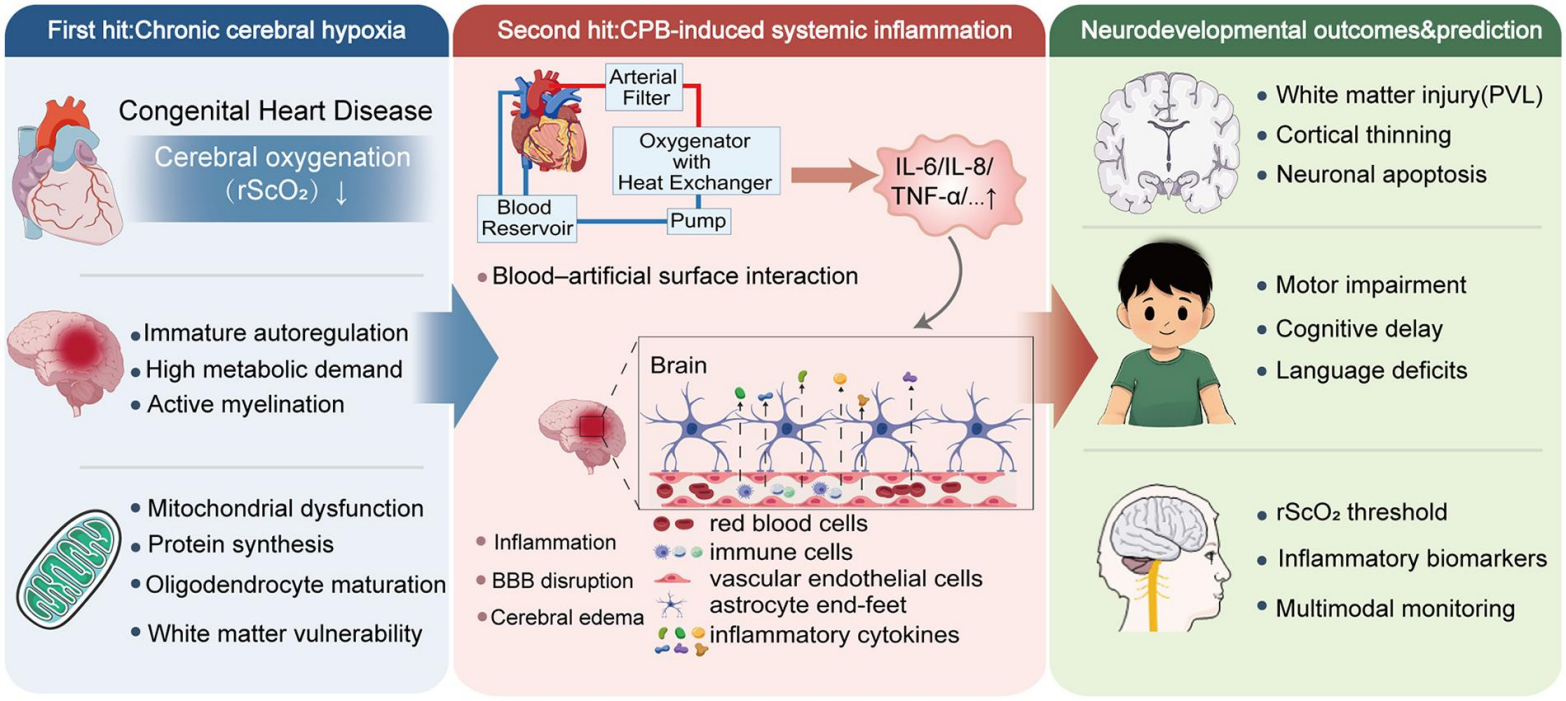
Conceptual two-hit framework for interpreting cerebral rScO_2_ abnormalities and neurological risk in children with CHD undergoing CPB. Children with CHD often have chronic preoperative cerebral hypoxia, leading to a vulnerable brain state characterized by impaired oxygen transport, mitochondrial dysfunction, and white matter fragility (first hit). CPB induces systemic inflammation, disrupts the blood-brain barrier, and activates microglia, synergistically amplifying pre-existing injury (second hit). Together, these processes lead to structural brain injury and adverse neurodevelopmental outcomes, including motor impairment, cognitive delay, and language deficits. Integrating perioperative rScO_2_ dynamics with inflammatory biomarkers and neuroimaging features may enable predictive models for early risk stratification and long-term outcome prediction. BBB, blood-brain barrier; IL-6, interleukin-6; IL-8, interleukin-8; MRI, magnetic resonance imaging; PVL, periventricular leukomalacia; rScO_2_, regional cerebral oxygen saturation; SIRS, systemic inflammatory response syndrome; TNF-α, tumor necrosis factor-α. Some graphical elements were adapted from Servier Medical Art (https://smart.servier.com/), licensed under CC BY 4.0 (https://creativecommons.org/licenses/by/4.0/). Some graphical elements were created with BioGDP materials (https://biogdp.com/). Additional elements were created by the authors. All components were assembled and finalized using Adobe Illustrator.

Chronic fetal and preoperative hypoxia places the developing brain in a highly vulnerable state at metabolic and structural levels, manifested by reduced cerebral oxygen saturation, adaptive downregulation of mitochondrial function, and impaired neurogenesis and myelination (59, 92). On this fragile background, CPB-induced systemic inflammation and neuroinflammatory cascades may act as a second hit, disrupting the blood–brain barrier, activating microglia, and amplifying hypoxic–ischemic injury, ultimately leading to gray and white matter damage and adverse neurodevelopmental outcomes (Figure 3). This two-hit model of chronic hypoxia combined with inflammation can help explain why preoperative structural abnormalities such as white matter injury persist after surgery and are closely associated with PVL and poor neurodevelopmental outcomes (57).

In addition, children with CHD commonly exhibit chronic systemic inflammation and immune dysfunction, which may be associated with multiple CHD-related and perioperative factors, including thymectomy, chronic hypoxia or venous congestion, genetic syndromes such as Down syndrome and DiGeorge syndrome, and comorbidities (93). These immune abnormalities may further modify vulnerability to perioperative cerebral oxygenation disturbances and influence the relationship between rScO_2_ changes and neurological outcomes.

## 6 Neurological injury and neurodevelopmental outcomes after CPB surgery for pediatric CHD

Existing studies examining the association between perioperative rScO_2_ dynamics after CPB and neurological or neurodevelopmental outcomes in children with CHD have primarily used prospective cohort, retrospective cohort, and observational designs (Supplementary Table S1). In this narrative review, Supplementary Table S1 is provided as a concise summary of representative studies rather than as a systematic evidence table; no formal risk-of-bias assessment or meta-analysis was performed. Because most available studies are observational, associations between low rScO_2_, desaturation burden, and adverse neurological or neurodevelopmental outcomes should not be interpreted as direct evidence of causality. Rather, perioperative rScO_2_ should currently be regarded primarily as a prognostic marker and risk-stratification tool. Whether active interventions that prevent or correct rScO_2_ abnormalities can improve long-term neurodevelopmental outcomes remains uncertain and requires adequately powered prospective interventional studies.

By longitudinally tracking perioperative cerebral oxygen parameters and subsequent neurological injury or neurodevelopmental scale scores, these studies suggest associations between abnormal cerebral oxygenation and long-term neurodevelopmental outcomes in real-world clinical settings and are feasible in infant and pediatric CHD populations (94, 95). In contrast, randomized controlled trials can actively intervene to optimize cerebral oxygenation (e.g., by adjusting CPB perfusion strategies), but their number remains limited in critical CHD populations due to ethical constraints and practical challenges (94). Regarding outcome measurement, neurocognitive assessment must be aligned with developmental stage. For infants and toddlers (≤2 years), the Bayley Scales of Infant and Toddler Development are commonly used, and the cognition, language, and motor subscales can quantify early development and risk of developmental delay (12, 94). For older children (>2 years), the Wechsler Preschool and Primary Scale of Intelligence (WPPSI) or Wechsler Intelligence Scale for Children (WISC) is often used to evaluate full-scale IQ and specific cognitive domains such as attention, executive function, and language (39, 57). To capture mid- to long-term effects of cardiac surgery on neurodevelopment, many studies perform standardized neurocognitive assessment at approximately 2 years postoperatively, when developmental stability is greater and the association between perioperative brain injury and later cognitive outcomes can be assessed more reliably.

Over the past decade (2016–2025), multiple studies (Supplementary Table S1) have further supported a close association between perioperative cerebral oxygen saturation and neurodevelopmental outcomes in children with CHD. In a cohort of children with hypoplastic left heart syndrome undergoing the Norwood procedure, higher preoperative rScO_2_ was associated with better IQ, whereas postoperative rScO_2_ showed no relationship with IQ. Specifically, mean preoperative rScO_2_ was lower in children with below-average cognitive scores, and preoperative rScO_2_ correlated positively with full-scale IQ, verbal IQ, and performance IQ (96). Spaeder et al. reported that lower postoperative rScO_2_ variability was associated with poorer neurodevelopmental outcomes, suggesting that the stability of cerebral oxygenation is important for neurodevelopment and that dynamic fluctuation patterns may reflect deeper physiological regulation (4). A Bayesian multivariable analysis showed that reduced mean rScO_2_ within the first 12–24 h postoperatively and a desaturation dose defined as rScO_2_ < 65% were independently associated with lower total IQ at 2 years (39), providing key evidence for postoperative neurodevelopmental risk surveillance and further supporting the prognostic value of cerebral oximetry.

Interpreting rScO_2_ together with metabolic indices may facilitate early identification of high-risk patients, providing a window to optimize perfusion, oxygen delivery and supportive strategies, and enabling early intervention before long-term neurodevelopmental outcomes become clinically apparent. Aly et al. similarly reported that low cTOI plus high lactate at 24 h was associated with death or neurodevelopmental impairment and proposed a combined threshold of cTOI < 58% and lactate >7.4 mmol/L, which had high sensitivity (95%) for predicting adverse outcomes (97). However, this finding should be interpreted in its original postoperative context. The association may reflect postoperative low-cardiac-output physiology, systemic oxygen-delivery failure, and lactate burden rather than a CPB-specific intraoperative neuromonitoring effect. Therefore, postoperative cTOI combined with lactate may help identify high-risk patients early, but it should not be interpreted as evidence that intraoperative NIRS-guided intervention improves neurological outcomes.

Multiple studies also suggest that brain injury biomarkers such as glial fibrillary acidic protein (GFAP), s100 calcium-binding protein B (S100B) and phosphorylated Tau protein (pTau) are associated with cerebral oxygen saturation and may serve as early indicators of neurodevelopmental impairment, although their associations with long-term outcomes require further validation. Hansen et al. found that increased postoperative S100B was associated with lower cerebral oxygen saturation. Notably, this association was observed in infants but not in neonates, suggesting age-related differences in biomarker sensitivity and potentially distinct injury pathways. Easley et al. found that impaired cerebral autoregulation during CPB was associated with increased GFAP, suggesting a risk of occult brain injury (98). Hansen et al. also found that the proportion of postoperative GFAP elevation was consistent with the incidence of white matter injury in neonates with CHD detected by previous MRI modalities (99). Lee et al. observed rapid increases in Ubiquitin C-terminal hydrolase L1 (UCHL1) in the DHCA group, supporting its potential as a candidate biomarker for acute brain injury associated with this surgical approach (99–101). Graham et al. reported that perioperative GFAP was associated with infant neurodevelopmental scores and may have potential for early risk stratification and therapeutic evaluation (102). In elective pediatric CHD surgery, Chiperi et al. combined NIRS-derived cerebral oxygen saturation with serum GFAP and other markers, assessed development using Denver Developmental Screening Test II scale (DDST-II) preoperatively and at 4–6 months postoperatively, and found that GFAP (especially in cyanotic CHD) had predictive value for short-term developmental impairment (103). In a prospective observational study of children with congenital heart disease undergoing cardiopulmonary bypass surgery, Chiperi et al. reported perioperative increases in serum pTau, showing a significant association with cerebral oxygen desaturation and predictive value for perioperative hypoxemia in cyanotic patients (104).

Furthermore, computational models are gradually integrating dynamic trends of rScO_2_ with clinical biomarkers and imaging information to enhance predictive performance. MRI metrics (e.g., brain volume, white matter connectivity, cortical thickness) can serve as tools for assessing neurodevelopmental outcomes (105). Zou et al. used the cerebral oximetry/pressure index (COPI) to evaluate cerebral autoregulation within 48 h after cardiac surgery in CHD children and found that impaired autoregulation was common, and COPI was associated with EEG/MRI injury severity and early outcomes (106). Kelly et al. also showed that reduced preoperative cerebral oxygen delivery in neonates with complex CHD correlated linearly with reduced cortical gray matter volume, providing imaging evidence for a mechanistic pathway linking chronic hypoxia, cortical dysmaturation and subsequent neurodevelopmental impairment (107). Lynch et al. found that during DHCA in CHD infants, rScO_2_ decreased exponentially with substantial inter-individual variability, and greater rScO_2_ decline was associated with higher risk of postoperative white matter injury on MRI (24). Importantly, imaging changes do not always correspond linearly to neurodevelopmental outcomes. Structural brain injury induced by abnormal cerebral oxygen metabolism may be occult, and its negative impact may not be readily detected by standardized scales in early infancy. Mueller et al. found in a study of patients with HLHS undergoing the comprehensive Stage II surgery that rScO_2_ was correlated with cerebral volume indices, but no significant association was observed with Bayley-III scores (108). De Silvestro et al. similarly reported that intraoperative desaturation was associated with perioperative brain structural alterations and new intracranial lesions but not with Bayley-III outcomes at 1 year (5). Therefore, neurodevelopmental evaluation after CHD surgery should be supported by a long-term and dynamic follow-up system that integrates neuroimaging metrics with age-appropriate functional scales to more comprehensively characterize long-term trajectories.

## 7 Research prospects of perioperative cerebral oxygen saturation in children with CHD—optimization of interventions and neuroprotection

### 7.1 Standardization of cerebral oximetry management protocols

In the clinical implementation of perioperative neuroprotection, NIRS has long been designated as a key bedside monitoring tool by multiple academic societies (109, 110). Evidence-based cerebral oximetry management protocols should integrate NIRS monitoring technology with the core concept of stratified intervention. During the preoperative phase, it is recommended to prioritize the acquisition of baseline rScO_2_ in children. Notably, baseline rScO_2_ levels in neonates with CHD are generally lower than those in healthy age-matched controls, and cerebrovascular stability is significantly affected by the anatomical and physiological subtypes of the disease (e.g., children with single-ventricle physiology are more prone to unstable fluctuations in cerebral oximetry) (111, 112). Therefore, the interpretation of baseline values must be closely combined with the anatomical classification of the disease and the underlying hemodynamic status.

For intraoperative and early postoperative management, the well-established concepts of risk thresholds and desaturation burden assessment from previous studies can be referenced. In the event of decreased cerebral oximetry after surgery, a tiered intervention protocol based on NIRS monitoring can be initiated: the first tier involves correcting reversible hemodynamic abnormalities (e.g., optimizing volume status, adjusting cardiac output or doses of vasoactive drugs); the second tier focuses on evaluating and optimizing oxygen delivery-related factors (e.g., adjusting ventilation parameters, optimizing oxygen concentration, correcting hemoglobin or hematocrit levels); if necessary, the third tier is implemented to investigate mechanical pathogenic factors (e.g., shunt-related complications). Existing studies have indicated that episodes of decreased cerebral oximetry detected by perioperative NIRS monitoring typically trigger the above-mentioned intervention protocols, yet there remain considerable variations in the setting of early warning thresholds and detailed intervention guidelines across different medical centers (47, 48, 113).

A survey of members of the Congenital Cardiac Anesthesia Society showed that 93.5% of respondents reported using tissue oximeters, while only 14.5% of their institutions had formal management protocols based on NIRS or tissue oxygenation (48). This suggests that the major bottleneck in current cerebral oximetry management may not be device accessibility, but rather the standardization of intervention trigger thresholds, management algorithms and follow-up endpoints.

### 7.2 Optimization of cerebral oximetry intervention strategies

During the cardiopulmonary bypass (CPB) phase, the core of goal-directed perfusion (GDP) is to ensure that oxygen delivery remains within a safe range, and to take pump flow, hematocrit, hemoglobin, arterial oxygen content and other variables as controllable levers for oxygen supply. This reduces the risk of perioperative cerebral hypoxic exposure and may mechanistically lower the incidence of long-term adverse neurodevelopmental outcomes. Recent machine learning-based studies have suggested that when the oxygen delivery indexed to body surface area (DO_2_i) is less than 350 mL/min/m^2^ during CPB in children, the risk of cardiac surgery-associated acute kidney injury is increased (114). Although the primary endpoint of this study was not cerebral injury, it demonstrates that oxygen delivery during pediatric CPB has a quantifiable threshold. GDP can link DO_2_i with lactate, venous oxygen saturation and rScO_2_ to form a strategy of tiered triggering and closed-loop correction. The latest prospective studies have shown that the cerebral autoregulation index can be calculated through the moving correlation analysis of mean arterial pressure and slow waves of cerebral oximetry, and based on this, the individualized optimal perfusion pressure target and the upper and lower limits of autoregulation can be derived (115). In the next few years, the most clinically feasible research direction for the perioperative period of children with CHD will be the clinical translation and validation of precise cerebral perfusion regulation and individualized targeted blood pressure management based on cerebral autoregulation. Oxygen supply and perfusion pressure should be simultaneously incorporated into the intervention bundle, with cerebral oxygen load and variability, cerebral MRI injury burden, postoperative epilepsy and EEG abnormalities selected as key intermediate endpoints. This will establish an association system between intervention measures and the neurodevelopmental outcomes of children at 6–24 months of age, school age and even in the long term. Existing randomized trials generally have a high risk of bias and considerable uncertainty in evidence. Whether maintaining a higher level of cerebral oximetry through active intervention can improve the long-term neurodevelopmental outcomes of children still requires further verification by higher-quality, multicenter randomized controlled trials (116). At present, rScO_2_ is better supported as a marker for risk stratification and physiological guidance than as an established modifiable therapeutic target. Whether preventing or correcting rScO_2_ abnormalities can improve long-term neurodevelopmental outcomes still requires validation in adequately powered, multicenter interventional studies.

In terms of drug and mechanism-targeted interventions, translational research focusing on the reperfusion-oxidative stress-neuroinflammation axis remains exploratory. Agents such as dexmedetomidine, nitric oxide, and allopurinol may provide biologically plausible research directions, but current evidence is insufficient to establish them as neuroprotective therapies for pediatric CHD surgery with CPB (75, 87, 91). Future studies should combine drug trials with multimodal monitoring (NIRS/EEG/MRI) and standardized neurodevelopmental follow-up to enhance the interpretability and translational relevance of mechanistic findings, intermediate phenotypes and long-term outcomes.

### 7.3 Research prospects for CPB-related cerebral injury

First, in the integration of multimodal monitoring, future research should actively explore the synergistic application of NIRS with electroencephalography, transcranial Doppler and other monitoring methods to more comprehensively characterize cerebral oxygen supply-demand balance and cerebral functional status. Data science and artificial intelligence need to move beyond outcome prediction to real-time decision support. Existing reviews have pointed out that a single NIRS index cannot fully reflect complex cerebral physiological changes, and multimodal monitoring is expected to improve the accuracy of risk identification and intervention timing judgment, but it still needs to be verified and standardized by prospective studies (12). Current multimodal models have attempted to integrate MRI features with physiological biomarkers and achieved a significant correlation in cognitive score prediction. Multimodal MRI combined with machine learning and deep learning technologies can realize accurate prediction of CHD-related cerebral injury through processes such as feature selection, model training and multimodal data fusion (117). Studies in the past 5 years have used machine learning methods to explore the key threshold of DO_2_i during pediatric CPB and evaluate its association with postoperative adverse outcomes (current evidence is mainly focused on CS-AKI). Meanwhile, newer studies have mostly focused on the early prediction of complications by integrating perioperative dynamic data with machine learning, but there is still a lack of high-quality validation studies centered on DO_2_i thresholds for cerebral injury outcomes (114, 118, 119).

Second, emerging model-based NIRS approaches may further extend cerebral oximetry beyond rScO_2_ trend monitoring toward quantitative assessment of cerebral hemodynamics and metabolism. NIRS-based modeling has been used to estimate cerebral oxygen extraction and cerebral metabolic rate of oxygen (CMRO_2_), although these estimates depend on model assumptions and physiological constraints (120). In parallel, optical monitoring of redox cytochrome-c-oxidase (rCCO/oxCCO) has been reviewed as a potential marker of mitochondrial oxygen metabolism (121). Building on these methodological developments, experimental pig models of cardiac arrest and cardiopulmonary resuscitation have applied model-based NIRS analysis to track cerebral microvascular and metabolic parameters, including oxygen diffusion, vascular volume fractions, and CMRO_2_-related changes (122). More recent hyperspectral NIRS work in the same experimental context further examined the relationship between CMRO_2_ and rCCO during cardiac arrest and resuscitation (123). Although these studies were not performed in pediatric CHD surgery, they illustrate how advanced NIRS methods may help distinguish oxygen delivery, oxygen extraction, and mitochondrial/metabolic responses, which could be relevant for future CPB-related brain-injury monitoring.

Third, biomarkers and multi-omics will drive the shift of CPB-related cerebral injury research from phenotypic description to molecular subtyping. Future research can continue to focus on mechanistic pathways such as mitochondrial dysfunction, microglia-mediated neuroinflammation and blood-brain barrier protection, and evaluate their intervenability. Relevant mechanistic evidence will also help screen for more specific biomarkers and multimodal indicators, and optimize risk stratification and the setting of intervention endpoints. Efforts should be made to develop and evaluate novel serum biomarkers for perioperative cerebral injury. In the meantime, a systematic review of metabolomics for CPB in infants with CHD proposed that targeted or untargeted metabolomics can help identify pathophysiological perturbations and discover potential therapeutic targets (124). It is recommended to establish perioperative biobanks and physiological signal repositories to form reusable multicenter research infrastructure for screening drug targets and predictive model features.

Fourth, at the level of neuroassessment processes and research methodology, strict control of confounding variables is the key to ensuring the reliability of research conclusions. Existing studies generally correct for potential confounding factors such as the anatomical complexity of CHD, preoperative cerebral injury status, CPB duration and family socioeconomic status through stratified analysis or multivariate statistical models (32, 39, 57). Although traditional multivariate regression models have been widely used in analysis, with the increasing complexity of research variables and outcome indicators, how to reasonably integrate prior knowledge into the models and improve the stability of parameter estimation remains an important methodological challenge. Conducting large-sample, multicenter prospective cohort studies and establishing standardized cerebral oximetry monitoring protocols and a unified neurodevelopmental assessment framework will become the key directions to optimize the efficiency of relevant assessments in the future (12, 125).

Finally, attention should also be paid to the issue of health equity. Multiple cohort studies from high-income countries have shown that the overall follow-up completion rate for neurodevelopment in children with CHD is relatively low, and socioeconomic factors (e.g., family economic status and educational opportunities, residential distance and healthcare accessibility) are key barriers affecting the persistence of follow-up. While optimizing oximetry management strategies, it is also necessary to improve follow-up support and resource allocation for vulnerable populations at the health system level (126–128).
