# Supplementary material for: Near-infrared spectroscopy cerebral oximetry in pediatric congenital heart disease with cardiopulmonary bypass: a narrative review of current evidence and neuroprotection
Source: Front Pediatr. 2026 Jul 8;14:1822134. doi: 10.3389/fped.2026.1822134 (PMC13388295; doi:10.3389/fped.2026.1822134)
Supplement: Supplementary file 1 [file Table1.docx]

Supplementary Table 1 Summary of key studies (2016-2025) examining the relationship between Cerebral O₂ and neurodevelopmental (ND) outcomes or brain injury in children with CHD

| **Study Design** | **Sample** | **Surgery Type** | **Cerebral O₂ Monitoring** | **ND Assessment** | **Key Findings** | **Ref.** |
| --- | --- | --- | --- | --- | --- | --- |
| Observational cohort | 86 children (3.5-7 years old, 43 with follow-up) | HLHS undergoing Norwood | NIRS (rScO₂) measured at 24h pre-op and 48h post-op | performance IQ scores were evaluated with the HAWIVA-III at a median of 4.5 years. Cognitive functions were assessed with the KET-KID | Mean pre-op rScO₂ was lower in children with below-average cognitive scores, and preoperative rScO₂ correlated positively with full-scale IQ (r = 0.495), verbal IQ (r = 0.524), and performance IQ (r = 0.386). A relationship between IQ or KET-KID scores and post-op rScO₂ was not found. | (1) |
| Observational case-control | 30 CHD neonates + 30 controls | Preoperative (before repair) | NIRS (rScO₂) pre-op | Neurodevelopmental surrogate via brain MRI cortical metrics (grey matter volume, gyrification index) | Reduced cerebral oxygen delivery correlated with impaired cortical development; lesions with lowest oxygen delivery showed greatest cortical dysmaturation. | (2) |
| Retrospective cohort | 62 neonates (44 with follow-up) | Neonatal CHD surgery under CPB | NIRS cerebral tissue O₂ index(cTOI), continuous peri-op; analyzed rScO₂ variability | BSID II, PDI MDI (at ~6, 15, 21 months) | Lower postoperative rScO₂ variability is associated with worse neurodevelopmental outcomes in neonates. Neonates with abnormal ND scores had significantly lower cerebral O₂ saturation variability post-op compared to those with normal scores. | (3) |

**Table 1.** *Cont.*

| **Study Design** | **Sample** | **Surgery Type** | **Cerebral O₂ Monitoring** | **ND Assessment** | **Key Findings** | **Ref.** |
| --- | --- | --- | --- | --- | --- | --- |
| Prospective  cohort | 75 neonates (40 single ventricle, 35 two-ventricle) (54 with follow-up) | Neonatal CHD surgery | NIRS cTOI at 1h and 24h post-CPB; | BSID II at ~6,5,21 months; plus survival tracked | Low cTOI combined with high lactate at 24 h postoperatively was associated with death or neurodevelopmental impairment. A combined threshold of cTOI <58% and lactate >7.4 mmol/L showed 95% sensitivity for predicting adverse outcomes. | (4) |
| Prospective, observational, multicenter | 57 children | Various CHD surgeries on CPB | NIRS-based autoregulation monitoring (HVx) | No ND follow-up (used GFAP as brain injury marker) | Impaired cerebral autoregulation during CPB correlated with elevated GFAP, suggesting silent brain injury risk. Individual dynamic HVx monitoring identified episodes of dysautoregulation linked to biomarker release. | (5) |
| Observational | 26 infants(< 3 months) | CHD with CPB (n = 12) or DHCA (n = 14) | NIRS rScO₂ during surgery | No ND follow-up (outcome = biomarker elevation(UCHL1 and pNF-H) levels 0,12,24,48h post-op | UCHL1 was rapidly elevated immediately postoperatively in the DHCA group, suggesting that it may serve as a potential biomarker for acute brain injury associated with this type of cardiac surgery; pNF-H did not show an association with postoperative brain injury. There was no correlation between biomarkers and NIRS at 0 hours. | (6) |

**Table 1.** *Cont.*

| **Study Design** | **Sample** | **Surgery Type** | **Cerebral O₂ Monitoring** | **ND Assessment** | **Key Findings** | **Ref.** |
| --- | --- | --- | --- | --- | --- | --- |
| Prospective observational cohort | 36 neonates (38 surgeries) | Neonatal complex CHD | NIRS rScO₂ during surgery; | No ND follow-up (outcome = biomarker elevation (GFAP) levels pre- and 0,12,24,48h post-op) | Elevated GFAP (astrocytic injury marker) in ~50% of neonates post-surgery was associated with lower cerebral saturations and older age at surgery, suggesting cerebral hypoperfusion/ischemia triggered astroglial injury. In arterial-switch patients, those with lower pre-op rScO₂ had higher GFAP after surgery. | (7) |
| Prospective observational cohort | 38 neonates, 56 infants(94 surgeries) | Neonatal CHD surgery | NIRS rScO₂ during surgery; | No ND follow-up (outcome = biomarker elevation, serum S100B levels pre- and 0,12,24,48h post-op) | 40.5% neonates and 41.8% infants had elevated S100B at 24/48h post-op. In infants, elevated S100B was significantly linked to perioperative cerebral oxygenation impairment (lower rScO₂, wider arterial-cerebral O₂ saturation difference), but not in neonates. Post-op S100B correlated positively with lactate in both groups. | (8) |
| Retrospective cohort | 19 infants | HLHS undergoing Comprehensive Stage II | NIRS rScO₂ during surgery | Brain MRI volumes + Bayley-III at 2–3 years | rScO₂ correlated with brain volumes (e.g., lower minima / higher AUC below 45% linked to smaller intracranial/white matter volumes), but no association with Bayley-III. | (9) |

**Table 1.** *Cont.*

| **Study Design** | **Sample** | **Surgery Type** | **Cerebral O₂ Monitoring** | **ND Assessment** | **Key Findings** | **Ref.** |
| --- | --- | --- | --- | --- | --- | --- |
| Prospective, longitudinal single-center | 12 neonates (<30 days) and 25 infants (30 days-6 months) | Pediatric CPB surgeries | NIRS Perioperative rScO₂ | No formal ND follow-up (used oxidative stress biomarker 8-iso-PGF2α), NBO | Pediatric cardiac surgery induces oxidative stress, which may relate to neurological outcomes. Significant increases in oxidative stress markers post-CPB, with possible associations between higher oxidative stress and lower intraoperative rScO₂.Global NBO score not associated with 8-iso-PGF2α descent; subdomain: inverse correlation with habituation. Age-adjusted. | (10) |
| Single-center prospective  observational | 87 children(<12 years) | Corrective or palliative CHD (majority with CPB; small DHCA subset) | Continuous NIRS rScO₂ | Total IQ at ~2-year follow-up using Wechsler tests (WPPSI-III-NL / WISC-III-NL) | Lower mean rScO₂ (especially first 12 h) and higher desaturation dose independently increased probability of lower total IQ at 2 years (Bayesian regression adjusted for confounders) | (11) |
| Cohort | 32 neonates | Critical CHD (majority d-TGA) | NIRS rScO₂ (Intraop) | Brain MRI (pre/post); Bayley-III at 1 year | Intraop cerebral desaturation associated with greater perioperative lateral ventricle volume change and more new intracranial lesions, but not associated with Bayley-III at 1 year | (12) |
| Prospective  observational | 80 children(<5 years) | CHD surgery after CPB | NIRS rScO₂ (48h post-op) | EEG + MRI brain injury; early outcomes (no long-term ND scale) | COPI associated with perioperative variables and brain injury severity; emphasizes blood pressure management early after CPB and calls for studies linking to long-term ND. | (13) |

**Table 1.** *Cont.*

| **Study Design** | **Sample** | **Surgery Type** | **Cerebral O₂ Monitoring** | **ND Assessment** | **Key Findings** | **Ref.** |
| --- | --- | --- | --- | --- | --- | --- |
| Prospective  observational | 42 children(<5 years, 13 cyanotic) | elective CHD surgery with CPB | NIRS rScO₂ (pre-, intra- and post-op) | DDST-II (pre-op; 4–6 months post-op) | No direct NIRS–DDST correlation reported; instead, in cyanotic CHD, GFAP showed a diagnostic model for significant NIRS variation (AUC≈0.70) and a prognostic model for developmental gain/impairment (AUC≈0.667) | (14) |
| Single-center, prospective observational pilot study | 40 children; age 0–6 years (non-cyanotic: n=27, cyanotic: n=13) | Predominantly corrective cardiac surgery; 2 palliative procedures; all with CPB | NIRS rScO₂.Cerebral desaturation defined as ≥20% decrease between max and min NIRS values | No ND follow-up (outcome = biomarker elevation, serum MBP and pTau levels pre- and 10-12 post-op); long-term follow-up ongoing) | Postoperative pTau and MBP levels increased in both groups (significant in non-cyanotic CHD); pTau associated with perioperative cerebral desaturation; in cyanotic CHD, pTau predicted cerebral oxygen reduction (ROC AUC = 0.70); MBP correlated with albumin, hemoglobin, height, and weight; NIRS values correlated with postoperative neuromarker levels | (15) |

Abbreviations: AUC = area under the curve; Bayley-III = Bayley scales of infant and toddler development, 3rd Edition; BSID II = Bayley scales of infant development, 2nd edition; CHD = congenital heart disease; COPI = Cerebral oximetry/pressure index; CPB = cardiopulmonary bypass; cTOI = cerebral tissue O₂ index; DDST-II = Denver Developmental Screening Test II scale; DHCA = deep hypothermic circulatory arrest; GFAP = glial fibrillary acidic protein; HAWIVA-III = Hannover-Wechsler intelligence scale, 3rd edition; HLHS = hypoplastic left heart syndrome; HVx = hemoglobin volume index; IQ = intelligence quotient; KET-KID = German Kognitiver Entwicklungstest für das Kindergartenalter; MDI = mental development index; MRI = magnetic resonance imaging; NBO = neonatal behavioral observations; PDI = psychomotor development index; pNF-H = phosphorylated axonal neurofilament heavy chain; pTau = phosphorylated Tau protein; S100B = s100 calcium-binding protein B; UCHL1 = ubiquitin c-terminal hydrolase L1; 8-iso-PGF2α = 8-isoprostaglandin F₂α.

**References**

1. Hansen JH, Rotermann I, Logoteta J, Jung O, Dutschke P, Scheewe J, et al. Neurodevelopmental Outcome in Hypoplastic Left Heart Syndrome: Impact of Perioperative Cerebral Tissue Oxygenation of the Norwood Procedure. *J Thorac Cardiovasc Surg* (2016) 151(5):1358-66. Epub 2016/04/18. doi: 10.1016/j.jtcvs.2016.02.035.

2. Kelly CJ, Makropoulos A, Cordero-Grande L, Hutter J, Price A, Hughes E, et al. Impaired Development of the Cerebral Cortex in Infants with Congenital Heart Disease Is Correlated to Reduced Cerebral Oxygen Delivery. *Sci Rep* (2017) 7(1):15088. Epub 2017/11/10. doi: 10.1038/s41598-017-14939-z.

3. Spaeder MC, Klugman D, Skurow-Todd K, Glass P, Jonas RA, Donofrio MT. Perioperative near-Infrared Spectroscopy Monitoring in Neonates with Congenital Heart Disease: Relationship of Cerebral Tissue Oxygenation Index Variability with Neurodevelopmental Outcome. *Pediatr Crit Care Med* (2017) 18(3):213-8. Epub 2017/01/10. doi: 10.1097/PCC.0000000000001056.

4. Aly SA, Zurakowski D, Glass P, Skurow-Todd K, Jonas RA, Donofrio MT. Cerebral Tissue Oxygenation Index and Lactate at 24 Hours Postoperative Predict Survival and Neurodevelopmental Outcome after Neonatal Cardiac Surgery. *Congenit Heart Dis* (2017) 12(2):188-95. Epub 2016/11/20. doi: 10.1111/chd.12426.

5. Easley RB, Marino BS, Jennings J, Cassedy AE, Kibler KK, Brady KM, et al. Impaired Cerebral Autoregulation and Elevation in Plasma Glial Fibrillary Acidic Protein Level During Cardiopulmonary Bypass Surgery for Chd. *Cardiol Young* (2018) 28(1):55-65. Epub 2017/08/25. doi: 10.1017/s1047951117001573.

6. Lee T, Chikkabyrappa SM, Reformina D, Mastrippolito A, Chakravarti SB, Mosca RS, et al. Ubiquitin C-Terminal Hydrolase 1 and Phosphorylated Axonal Neurofilament Heavy Chain in Infants Undergoing Cardiac Surgery: Preliminary Assessment as Potential Biomarkers of Brain Injury. *World J Pediatr Congenit Heart Surg* (2018) 9(4):412-8. Epub 2018/06/28. doi: 10.1177/2150135118762390.

7. Hansen JH, Kissner L, Chitadze G, Logoteta J, Jung O, Dütschke P, et al. Glial Fibrillary Acid Protein and Cerebral Oxygenation in Neonates Undergoing Cardiac Surgery. *The Thoracic and cardiovascular surgeon* (2019) 67(S 04):e11-e8. Epub 2020/01/02. doi: 10.1055/s-0039-3401793.

8. Hansen JH, Kissner L, Logoteta J, Jung O, Dütschke P, Attmann T, et al. S100b and Its Relation to Cerebral Oxygenation in Neonates and Infants Undergoing Surgery for Congenital Heart Disease. *Congenit Heart Dis* (2019) 14(3):427-37. Epub 2019/01/04. doi: 10.1111/chd.12741.

9. Mueller M, Zajonz T, Mann V, Koerner C, Akintuerk H, Yoerueker U, et al. Interrelations of Intraoperative Changes in Cerebral Tissue Oxygen Saturation with Brain Volumes and Neurodevelopment Outcome after the Comprehensive Stage Ii Procedure in Infants with Hypoplastic Left Heart Syndrome: A Retrospective Cohort Study. *J Cardiothorac Vasc Anesth* (2021) 35(10):2907-12. Epub 2021/01/13. doi: 10.1053/j.jvca.2020.12.013.

10. Hadley S, Cañizo Vazquez D, Lopez Abad M, Congiu S, Lushchencov D, Camprubí Camprubí M, et al. Oxidative Stress Response in Children Undergoing Cardiac Surgery: Utility of the Clearance of Isoprostanes. *PloS one* (2021) 16(7):e0250124. Epub 2021/07/07. doi: 10.1371/journal.pone.0250124.

11. Carra G, Flechet M, Jacobs A, Verstraete S, Vlasselaers D, Desmet L, et al. Postoperative Cerebral Oxygen Saturation in Children after Congenital Cardiac Surgery and Long-Term Total Intelligence Quotient: A Prospective Observational Study. *Critical care medicine* (2021) 49(6):967-76. Epub 2021/02/17. doi: 10.1097/ccm.0000000000004852.

12. De Silvestro AA, Kruger B, Steger C, Feldmann M, Payette K, Kruger J, et al. Cerebral Desaturation During Neonatal Congenital Heart Surgery Is Associated with Perioperative Brain Structure Alterations but Not with Neurodevelopmental Outcome at 1 Year. *Eur J Cardiothorac Surg* (2022) 62(5). Epub 2022/04/05. doi: 10.1093/ejcts/ezac138.

13. Zou M, Yu L, Lin R, Feng J, Zhang M, Ning S, et al. Cerebral Autoregulation Status in Relation to Brain Injury on Electroencephalogram and Magnetic Resonance Imaging in Children Following Cardiac Surgery. *J Am Heart Assoc* (2023) 12(12):e028147. Epub 2023/06/11. doi: 10.1161/jaha.122.028147.

14. Chiperi LE, Hutanu A, Tecar C, Muntean I. Serum Markers of Brain Injury in Pediatric Patients with Congenital Heart Defects Undergoing Cardiac Surgery: Diagnostic and Prognostic Role. *Clin Pract* (2023) 13(5):1253-65. Epub 2023/10/27. doi: 10.3390/clinpract13050113.

15. Chiperi LE, Tecar C, Hutanu A. Serum Tau Protein and Myelin Basic Protein in Pediatric Patients with Congenital Heart Defects Undergoing Cardiac Surgery: Preliminary Assessment as Novel Neuromarkers of Brain Injury. *Ir J Med Sci* (2024) 193(3):1229-37. Epub 2023/12/17. doi: 10.1007/s11845-023-03582-5.
